# Higher Diplomas

**Published:** 1921-08-27

**Authors:** 


### HIGHER DIPLOMAS.

**Royal College of Physicians of London** grants its membership after examination to graduates in medicine of recognised Universities, or to licentiates of the College, being above the age of twenty-five years, who do not engage in trade, do not dispense medicine, and who do not practise in partnership. The examination, which is held in January, April, July, and October, is partly written and partly oral. The fee for the membership is £42, but if the candidate is a licentiate the fee is the difference between what he has already paid for the licence and £42. In either case £6 6s. is paid before examination. The licence is now only issued through the Conjoint Board; for particulars apply to the Examination Hall, Queen Square, W.C. 1. The Fellowship is elective.

**Royal College of Surgeons of England.**—Candidates are required to complete five years of professional study after passing a recognised preliminary examination in general education. The diploma of membership is issued through the Conjoint Board in conjunction with the licence of the Royal College of Physicians of London. (See above.)

**Royal College of Physicians of Edinburgh** admits to its membership any licentiate of a British or Irish College of Physicians, or graduate in medicine of a University within the British Empire, approved by the Council, over twenty-four years of age, after examination in medicine and therapeutics and one or more of the following subjects selected by the candidate : (1) One or more departments of medicine specially professed, (2) psychological medicine, (3) general pathology and morbid anatomy, (4) medical jurisprudence, (5) public health, (6) midwifery, (7) diseases of women, (8) diseases of children, (9) tropical medicine. The fee to be paid by a licentiate is 20 guineas, by others 35 guineas. A member of three years' standing may be elected a Fellow. The fee is £64 18s.

**Royal College of Surgeons of Edinburgh.**—The Fellowship is granted, after examination, to any registered practitioner (a male or female), who is twenty-five years of age, and has been in practice for two years, on petition being granted. The examinations are written, oral, and practical. The fee is £35 to those who hold the diploma of licentiate of the College, and £45 to others (no stamp duty is payable on the diploma). Apply for particulars to Mr. D. L. Eadie, 49 Lauriston Place, Edinburgh.

**Royal Faculty of Physicians and Surgeons of Glasgow.**—Registered practitioners are admitted to the Fellowship by examination and by subsequent election. Four examinations are held annually (January, April, July, October). Fourteen days' notice must be given. The fee is £30 unless the candidate desires to qualify to hold office, when it is £50. In the case of a licentiate of the faculty, £25 and £15 respectively.

**Royal College of Surgeons in Ireland Fellowship.**— The examination for the Fellowship is divided into two parts—namely, the primary and the final. The final includes the examination of patients and the performance of operations on the dead body. Candidates are required to lodge their applications, certificates, and receipts for fees with the Registrar at least fourteen days before the date of the examination.

